# Systematic review of neuropsychological instruments used in subthalamic nucleus deep brain stimulation in Parkinson´s disease patients

**DOI:** 10.1590/1980-57642018dn13-020004

**Published:** 2019

**Authors:** Eduarda Naidel Barboza e Barbosa, Helenice Charchat-Fichman

**Affiliations:** 1 Master, Pontifical Catholic University of Rio de Janeiro (PUC-Rio), Rio de Janeiro, RJ, Brazil.; 2 Professor, Pontifical Catholic University of Rio de Janeiro (PUC-Rio), Rio de Janeiro, RJ Brazil.

**Keywords:** Parkinson’s disease, deep brain stimulation, neuropsychological instruments, neuropsychological assessment, doença de Parkinson, estimulação cerebral profunda, instrumentos neuropsicológicos, avaliação neuropsicológica

## Abstract

In addition to drug treatment, surgical intervention represents an alternative to PD patients with motor deficits. The most common intervention is subthalamic nucleus deep brain stimulation (STN-DBS). It is extremely important to perform a neuropsychological assessment in patients with STN-DBS, not only to identify losses related to the disease, but also to compare influence on cognition both pre and postoperatively.

**Objective:**

the objective of this systematic review was to investigate the instruments frequently used in studies related to STN-DBS in PD patients.

**Methods:**

articles were retrieved from Medline/Pubmed databases published in the 2007-2017 period using PRISMA criteria.

**Results:**

after analyzing 27 articles, the absence of a specific evaluation protocol for PD with STN-DBS was evident.

**Conclusion:**

non-motor symptoms are not given due importance in neuropsychological assessments. It is crucial to acknowledge that these symptoms have a major impact on the quality of life of patients. Greater engagement in assessing these aspects is required, in order to bridge the gaps in research.

## RESUMO.

Além do tratamento medicamentoso, a intervenção cirúrgica é uma alternativa aos pacientes com DP com déficits motores. A mais comum é a estimulação cerebral profunda do núcleo subtalâmico (ECP-NST). É extremamente importante realizar uma avaliação neuropsicológica em pacientes com ECP-NST, não apenas para identificar perdas relacionadas à doença, mas também para comparar a influência na cognição em cirurgias pré e pós-operatórias.

**Objetivo:**

esta revisão sistemática teve como objetivo investigar os instrumentos frequentemente utilizados em pesquisas relacionadas a ECP-NST em pacientes com DP.

**Métodos:**

Os artigos foram coletados nas bases de dados Medline / Pubmed publicadas no período de 2007-2017, utilizando os critérios do PRISMA.

**Resultados:**

após a análise de 27 artigos, percebeu-se a ausência de um protocolo de avaliação específico para a DP com ECP-NST.

**Conclusão:**

os sintomas não motores não tem recebido a devida importância na avaliação neuropsicológica. É fundamental reconhecer que eles representam grande influência na qualidade de vida dos pacientes. É necessário maior engajamento na avaliação desses aspectos, a fim de preencher as lacunas das pesquisas.

Parkinson’s disease (PD) is considered the second-most-common neurodegenerative disease, preceded only by Alzheimer’s disease (AD).^1^^,^ 2 PD’s motor characteristics are much better known than its non-motor ones, but patients also have functional impairment.^1^^,^ 3 ^-^ 5 When PD was first described, cognition was believed to be preserved, but current research^6^^,^ 7 reports cognitive decline. Besides drug treatment, surgical intervention can be used in some cases. One of these methods is deep brain stimulation (DBS), consisting of electrical stimulation of subcortical structures. The main objective of DBS is motor control of symptoms; however, the stimulated areas are also potentially able to stimulate some cognitive functions secondarily.^8^

Studies usually promote cognitive screening in patients to characterize the sample and identify the impairments to be analyzed. However, comparing cognitive data from different populations and based on different tests can produce conflicts in the literature, mainly because some screening instruments do not provide the sensitivity to assess cognitive functioning sufficiently^5^, while others employ different versions of the same test or use non-standard tasks.

The objective of this review was to learn about and understand the use of some instruments used in studies of PD patients with STN-DBS and to relate these findings with the literature in general. The search included articles published between January 2007 and January 2017, based on The Preferred Reporting Items for Systematic Review and Meta-Analyses (PRISMA) criteria.

## METHODS

The systematic review is a type of scientific study that aims to gather, critically evaluate and produce a synthesis of multiple primary studies.^9^

### Bibliographic survey

We designedasystematic review of the literature according to the Preferred Reporting Items for Systematic Review and Meta-Analyzes (PRISMA) criteria. The following terms were used: “Deep Brain Stimulation”, “DBS”, “Cognitive Functions” and “Parkinson Disease” with the Boolean operator “AND”. We selected scientific papers published in English between January 2007 and January 2017, involving comparative clinical trials in humans, from the Medline/Pubmed databases. Articles published before 2007, systematic reviews, case studies, book chapters and studies using animals were excluded.

### Study selection

**Figure 1:**
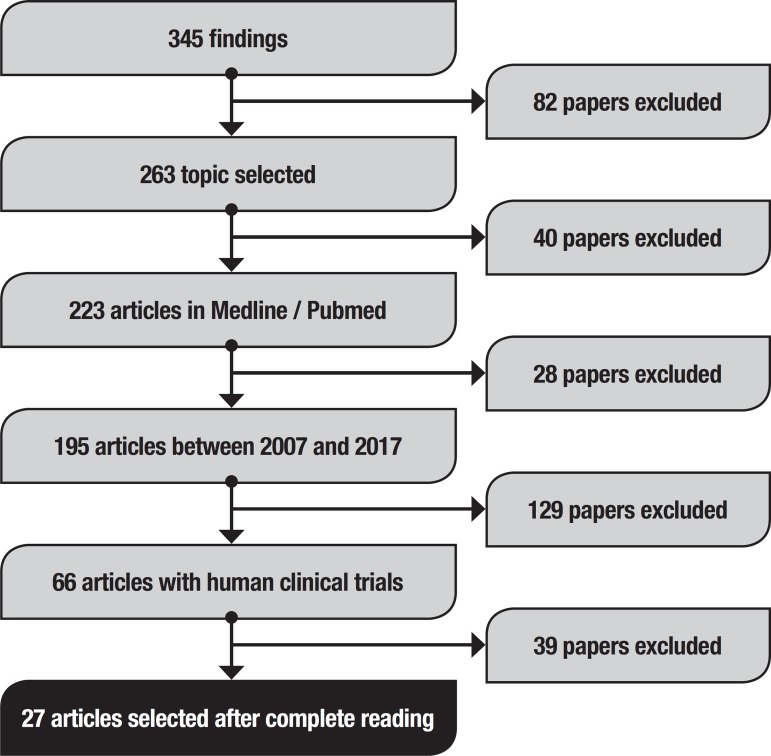
Article search flow diagram.

Initially, this method retrieved 345 studies (Figure 1). To refine the search, the following topics were selected: “Parkinson’s Disease”, “Subthalamic Nucleus”, “Deep Brain Stimulation”, “DBS”, “Cognition” (263), published on the Medline/Pubmed database (223) between 2007 and 2017 (195). From the material retrieved, we examined titles and abstracts for studies involving only human clinical trials (66). Literature reviews and case studies were excluded, as were articles with problems in the methodology, such as absence of (a) inclusion and exclusion criteria, (b) complete assessment protocol and (c) pre or post-surgery assessment (27). The researchers selected the articles independently: considering suitable studies that (a) evaluated PD patient cognition with STN-DBS; (b) reported the instruments and domains evaluated; and (c) presented pre and post-surgical results.

## RESULTS

The final list of articles included based on the search criteria in order of year, with Objectives and Results (Table 1), a list of instruments with quantity, separated by domains (Table 2) and a list of instruments used before and after DBS implantation to assess the cognitive aspects of the patients (Table 3) are given below.

**Table 1:** List of articles included in the systematic review criteria.

	Authors and name	Year	Instruments used
**1**	Cilia et ai. Brain networks underlining verbal fluency decline during STN-DBS in Parkinson’s disease: An ECD-SPECT study.	2007	Mini-Mental State Exam (MMSE), Phonemic and Semantic Verbal Fluency Tasks, Wisconsin Card Sorting Test (WCST), Raven's Progressive Matrices (RPM)
**2**	Klempirova et al. Deep brain stimulation of the subthalamic nucleus and cognitive functions in Parkinson’s disease.	2007	Mattis Dementia Rating Scale (MDRS), Wechsler Memory Scale-Ill (WMS), Stroop Test, VFT
**3**	Castelli et al. Apathy and verbal fluency in STN-stimulated PD patients.	2007	RPM, Bi-Syllabic Words Repetition test (BWR), Corsi's Block Tapping test (CBTT), WMS, Trail Making Test (TMT), Nelson Modified Card Sorting test (MCST), VTF, Beck Depression Inventory (BDI), Apathy Evaluation Scale (AES)
**4**	Heo et al. The effects of bilateral Subthalamic Nucleus Deep Brain Stimulation (STN-DBS) on cognition in Parkinson disease.	2008	TMT, Korean Boston Naming test (K-BNT), Rey-Kim Memory Battery, Grooved pegboard test, WCST, Stroop test, VFT, Korean Mini-Mental Status Examination (K-MMSE), BDI
**5**	Witt et al. Neuropsychological and psychiatric changes after deep brain stimulation for Parkinson’s disease: a randomised, multicentre study.	2008	UPDRS-I MDRS, German Rey's Auditory Verbal Learning Test (G-RAVLT), Wechsler Adult Intelligence Scale (WAIS), Benton Visual Retention Test, Stroop test, VFT, BDI, Montgomery- Asberg Depression Rating Scale (MADRS), Beck Anxiety Inventory (BAI), Parkinson's Disease Questionnaire (PDQ-39)
**6**	Alberts et al. Bilateral subthalamic stimulation impairs cognitive-motor performance in Parkinson’s disease patients.	2008	N-backtask, dual task
**7**	Lueken et al. Impaired performance on the Wisconsin Card Sorting Test under left- when compared to right-sided deep brain stimulation of the subthalamic nucleus in patients with Parkinson’s disease.	2008	MMSE, WCST, German Hospital Anxiety and Depression Scale (HADS-D), German Apathy Evaluation Scale (AES) *
**8**	Zangaglia et al. Deep brain stimulation and cognitive functions in Parkinson’s disease: A three-year controlled study	2009	MMSE, long memory task, verbal span, digit span, CBTT, WCST, RPM, FVT
**9**	York et al. Relationship between neuropsychological outcome and DBS surgical trajectory and electrode location	2009	MMSE, RAVLT, VFT, MDRS, BDI, State-Trait Anxiety Inventory (STAI)
**10**	Williams et al. Deep brain stimulation plus best medical therapy versus best medical therapy alone for advanced Parkinson’s disease.	2009	UPDRS-I, PDQ-39, MDRS, Delis-Kaplan executive function system (D-KEFS), Wechsler Abbreviated Scale Intelligence (WASI)
**11**	Daniels et al. Risk factors for executive dysfunction after subthalamic nucleus stimulation in Parkinson’s disease	2010	MDRS, RAVLT, WAIS, BVRT, Stroop test, VFT
**12**	Castelli et al. Neuropsychological changes 1-year after subthalamic DBS in PD patients: A prospective controlled study	2010	RPM, Bi-Syllabix Words Repetition Test, Corsi's Block Tapping test, Paired Associate Learning, Trail Making Test, MCST, VFT
**13**	Fasano et al. Motor and cognitive outcome in patients with Parkinson’s disease 8 years after subthalamic implants	2010	MMSE, Corsi's Block Tapping Test, digit span forward, digit span backward, RAVLT, RPM, MWCST, VFT, Zung's depression Scale, Zung's Anxiety Scale
**14**	Van Wouwe et al. Deep Brain Stimulation of the Subthalamic Nucleus Improves Reward- Based Decision-Learning in Parkinson’s Disease	2011	Flaruno and Kawato Task, MMSE
**15**	Israeli-Korn et al. Subthalamic Nucleus Deep Brain Stimulation Does Not Improve Visuo- Motor Impairment in Parkinsons Disease	2013	MMSE, BDI, VFT, Frontal Assessment Battery (FAB), Visual Analog Mood Scale, Digit Span Forward and Digit Span Backward, Finger Tapping Test, Visual-motor Coordination Task
**16**	Kim et al. Initial cognitive dip after subthalamic deep brain stimulation in Parkinson disease	2013	MMSE, TMT, K-BNT, Rey-Kim Memory Battery, Stroop Test, VFT, BDI
**17**	Yagiiez et al. Cognitive predictors of cognitive change following bilateral subthalamic nucleus deep brain stimulation in Parkinson’s disease	2014	WAIS-III, Recognition Memory Test, Birt Memory and Information Processing Battery, Graded Naming Test, Visual Object and Space Perception Battery, Hayling Sentence Completion Test, Brixton Spatial Anticipation Test, VFT
**18**	Asahi et al. Impact of bilateral subthalamic stimulation on motor/cognitive functions in Parkinson’s disease	2014	MMSE, Japanese Adult Reading Test (JART), Repeatable Battery for the Assessment of Neuropsychological Status (RBANS), WAIS-Revised
**19**	Rizzone et al. Long-term outcome of subthalamic nucleus DBS in Parkinson’s disease: From the advanced phase towards the late stage of the disease?	2014	UPDRS-I, MMSE, RPM, Digit Span Forward, Corsi's Block Test, MWCST, VFT, RAVLT, TMT, Paired Associated Learning, Attentive Matrices, Zung's Depression Scale, Zung's Anxiety Scale, BDI, State-Trait Anxiety Inventory (STAI)
**20**	Houvenaghel et al. Reduced Verbal Fluency following Subthalamic Deep Brain Stimulation: A Frontal-Related Cognitive Deficit?	2015	MDRS, VFT, Stroop Test, TMT, MCST, MADRS, Apathy Evaluation Scale (AES)
**21**	Markser et al. Deep brain stimulation and cognitive decline in Parkinson’s disease: The predictive value of electroencephalography	2015	MMSE, MDRS, Dem-Tech
**22**	Pham et al. Self-Reported Executive Functioning in Everyday Life in Parkinson’s Disease after Three Months of Subthalamic Deep Brain Stimulation	2015	MDRS, Behavior Rating Inventory of Executive Function-Adult Version (BRIEF-A), Symptom Checklist 90 - Revised (SCL-90-R), AES
**23**	Tang et al. Evidence of improved immediate verbal memory and diminished category fluency following STN-DBS in Chinese-Cantonese patients with idiopathic Parkinson’s disease	2015	Montreal Cognitive Assessment Hong Kong version (HK-MoCA), Chinese Auditory Verbal Learning Test (CAVLT), BVRT, Chinese modified version of BNT, Hooper Visual Organization Test (HVOT), Stroop Test Chinese Victoria version, VFT (semantic), BDI-II, BAI
**24**	Tremblay et al. The effects of subthalamic deep brain stimulation on metaphor comprehension and language abilities in Parkinson’s disease	2015	MoCA, Metaphor Comprehension Task, VFT (semantic), Alternation VFT, Lexical Decision Test, Word Association Test, BDI version IA
**25**	Krishnan et al. The decade after subthalamic stimulation in advanced Parkinson’s disease: A balancing act.	2016	UPDRS-I, MMSE, Addenbrooke's Cognitive Examination, BDI, Parkinson's Disease Quality of Life (PDQL) Questionnaire
**26**	Vonberg et al. Fabian. Deep Brain Stimulation of the Subthalamic Nucleus Improves Lexical Switching in Parkinson’s Disease Patients	2016	Parkinson Neuropsychometric Dementia Assessment (PANDA), German VFT
**27**	Ventre-Dominey et al. Distinct effects of dopamine vs STN stimulation therapies in associative learning and retention in Parkinson disease	2016	Conditional Associative Learning (CAL), Visual spatial Working Memory Task, Non-spatial Working Memory Task

**Table 2:** List and frequency of instruments used by domain.

N°	Domains Assessed	Instruments	N° of articles
**1**	**Activities of Daily Living**	UPDRS-I Non-Motor Experiences	3
**2**	**PD Quality of Life**	Parkinson's Disease Questionnaire (PDQ-39)	2
		Parkinson's Disease Quality of Life (PDQL)	1
3	**Global Functioning**	Mini-Mental State Exam (MMSE)*	14
		Mattis Dementia Rating Scale (MDRS)	8
		Raven's Progressive Matrices (RPM)	6
		Wechsler Adult Intelligence Scale (WAIS-III)*	4
		Montreal Cognitive Assessment (MoCA)*	2
		Addenbrooke's Cognitive Examination	1
		Japanese Adult Reading Test (JART)	1
		Repeatable Battery for the Assessment of Neuropsychological Status (RBANS)	1
		Dem-Tech	1
		Parkinson Neuropsychometric Dementia Assessment (PANDA)	1
**4**	**Psychiatric Symptoms**	Brief Psychiatric Rating Scale (BPRS)	1
		Visual Analogue Mood Scale	1
		Symptom Checklist 90 - Revised (SCL-90-R)	1
**5**	**Executive Functioning**	Verbal Fluency Tasks - Semantic*	19
		Verbal Fluency Tasks - Phonemic*	17
		Wisconsin Cards Sorting Test (WCST)*	9
		Stroop Test*	7
		Trail Making Test (TMT)*	6
		Digit Span Forward and Backward	5
		Frontal Assessment Battery (FAB)	1
		Behavior Rating Inventory of Executive Function - Adult Version (BRIEF-A)	1
		Hayling Sentence Completion Test	1
		Brixton Spatial Anticipation Test	1
		Haruno and Kawato Task (2006)**	1
		Delis-Kaplan Executive Function System (D-KEFS)	1
		N-back and dual task	1
		Visual Spatial and Non-spatial Working Memory Task	1
**6**	**Memory**	Rey's Auditory Verbal Learning Test (RAVLT)*	6
		Corsi's Block Tapping Test (CBTT)	5
		Paired Associate Learning (Wechsler Memory Scale)	4
		Bi-Syllabic Words Repetition test (BWR)	2
		Rey-Kim Memory Battery	2
		Recognition Memory Test	1
		Birt Memory and Information Processing Battery	1
		Long Memory Task and Verbal Span	1
		Conditional Associative Learning (CAL)**	1
**7**	**Language**	Boston Naming Test (BNT)*	3
		Graded Naming Test	1
		Metaphor Comprehension Task	1
		Lexical Decision Test	1
		Word Association Test	1
**8**	**Language**	Attentive Matrices	1
**9**	**Perception**	Incomplete Letters and Object Decision tasks (Visual Object and Space Perception Battery)	1
**10**	**Visuospatial Skills**	Benton Visual Retention Test	3
		Hooper Visual Organization Test (HVOT)	1
**11**	**Mood**	Beck Depression Inventory (BDI)	10
		Apathy Evaluation Scale (AES)*	4
		State-Trait Anxiety Inventory (STAI)	2
		Zung's Anxiety Scale	2
		Beck Anxiety Inventory (BAI)	2
		Zung's Depression Scale	2
		Montgomery-Asberg Depression Rating Scale (MADRS)	2
		Hospital Anxiety and Depression Scale (HADS-D)*	1
		Snaith-Hamilton Pleasure Scale	1
		Bech-Rafaelsen Mania Scale	1
**12**	**Sensory-Motor** **Coordination**	Grooved Pegboard Test	1
		Visual-motor Coordination Task	1
		Finger Tapping Test	1

**Table 3:** Instruments most used pre and post-DBS.

Instruments	
• Parkinson's Disease Questionnaire (PDQ-39)	
• Parkinson's Disease Quality of Life (PDQL)	
• Mini-Mental State Exam (MMSE)*	
• Mattis Dementia Rating Scale (MDRS)	
• Raven's Progressive Matrices (RPM)	
• Symptom Checklist 90 - Revised (SCL-90-R)	
• Verbal Fluency Tasks - Semantic*	
• Verbal Fluency Tasks - Phonemic*	
• Wisconsin Cards Sorting Test (WCST)*	
• Stroop Test*	
• Trail Making Test (TMT)*	
• Digit Span Forward and Backward	
• Rey's Auditory Verbal Learning test (RAVLT)*	
• Corsi's Block Tapping test (CBTT)	
• Rey-Kim Memory Battery	
• Boston Naming Test (BNT)*	
• Attentive Matrices	
• Incomplete Letters and Object Decision tasks	
• (Visual Object and Space Perception Battery)	
• Benton Visual Retention Test	
• Beck Depression Inventory (BDI)	
• Apathy Evaluation Scale (AES)*	
• State-Trait Anxiety Inventory (STAI)	
• Beck Anxiety Inventory (BAI)	
• Grooved Pegboard Test	

## DISCUSSION

A total of 61 (sixty-one) instruments were used to evaluate different aspects of patients, including batteries, subtests, scales and tasks (Table 2). These can be ordered from the most evaluated to least used, as follows: executive functions (14), global cognitive functioning (10) and mood (10), memory (9), language (5), psychiatric symptoms (3) and sensory-motor coordination (3), patient quality of life (2) and visuoconstructive skills (2) and attention (1), perception (1) and activities of daily living (1). Early in the onset of symptoms, 24% of patients presented cognitive impairment, especially memory problems, and executive function disorders: selective attention, flexibility in reasoning and planning capacity, visuoconstructive skills and naming ability.^9^

What justifies the most evaluated domains in the selected articles? PD patients with mild cognitive impairment (MCI), compared with PD patients without MCI, have significantly poorer performance on almost all cognitive domains: executive functions, attention, memory, and language.^7^ One in three patients with PD has cognitive impairment at the time of, or shortly after, diagnosis, progressively worsening or even causing dementia in the advanced stages.^6^ However, cognitive alterations are common even in non-PD patients.^10^ Cognitive impairment increases the risk of dementia, by 1.7 to 5.9, and early detection and identification of dementia risk is a major challenge due to the heterogeneity of patient profiles.^6^ The prevalence of dementia in PD is 24 to 31%,^11^ thus, evaluating the PD patient in a global and continuous way is the best path for monitoring the evolution of the effects of the disease. Comorbidity with dementia can be justified when we consider the ascending involvement of the brainstem to the cortical area. Microscopic modifications may be incorporated into its pathophysiology, including losses of neurons, gliosis, while surviving neurons may contain Lewy bodies. The loss of neurons markedly affects the substantia nigra, although it is not restricted to it. The damage also affects the aminergic nuclei of the brainstem, Meynert’s basal nucleus, hypothalamic nuclei and olfactory bulb.^12^ For this reason, it is essential to investigate the effects of surgery, such as STN-DBS, on the different aspects of a subject with PD.

The MMSE was the most used instrument^13^^-^ 25 for assessing global cognitive functioning among the selected studies. It was followed by the MDRS^17^^,^ 24 ^,^ 26 ^-^ 31 and RPM.^13^^,^ 16 ^,^ 18 ^,^ 32 The MMSE has several favorable qualities such as fast administration, easy interpretation for use during medical consultation; patient acceptability; cultural independence; and both language and education, which makes it easier to reproduce in different studies and provides similar performance among examiners. In contrast, the instrument is influenced by subjective or non-standardized application and interpretation by professionals. Screening tests, such as these, known and widely used, are highly dependent on a minimal educational level and have low sensitivity and specificity.^33^ Thus, an evaluation protocol containing only this instrument to evaluate global cognitive functioning would have little range in terms of the patient´s cognitive loss.

Several instruments were used to assess the EF of PD patients with STN-DBS, but the most recurrent were verbal fluency tasks, both semantic and phonemic,^13^^,^ 14 ^,^ 16 ^-^ 18 ^,^ 20 ^,^ 21 ^,^ 23 ^,^ 26 ^,^ 27 ^,^ 29 ^,^ 30 ^,^ 32 ^,^ 34 ^,^ 35 ^,^ 37 ^,^ 38 followed by the WCST.^13^^-^ 16 ^,^ 18 ^,^ 23 ^,^ 30 ^,^ 32 ^,^ 34 These tasks, in particular, underwent different adaptations in each article. Evaluating EFs is a major challenge, as is defining the concept. In general, it is understood as abilities that involve planning, organization, flexibility, monitoring and inhibitory control,^39^ presenting an adaptive value for the subject, since their performance on activities related to personal, professional and other domains also become impaired.^40^ Executive dysfunction is not always associated with memory, language or visuospatial skill impairment, among others, but rather a functional decline that can often be assessed based on self-report or a caregiver and/or family member informant. In patients with PD, it is a predictor of impairment, leading to ADL deficits.^8^

A number of studies^14^^,^ 16 ^-^ 18 ^,^ 21 ^,^ 23 ^,^ 27 ^,^ 29 ^,^ 32 ^,^ 34 ^,^ 35 ^,^ 36 ^,^ 41 used 9 different types of tests to evaluate memory, predominantly the RAVLT (memory and verbal learning) and CBTT (memory and visual learning). The neocortex and striatum are structures involved in implicit memory processing and dopamine is the neurotransmitter involved in the formation of these memories.^2^^,^ 42 Therefore, with the dopaminergic deficit and degeneration of the basal nuclei involved in PD pathology, this processing and pre-activation of the priming and learning procedures are impaired. In the early stages of PD, there are deficits in the implicit learning of new tasks. Implicit learning is the process through which we become sensitive to certain regularities in the environment, in the absence of the intention to learn about these same regularities and in such a way that the resulting knowledge is difficult to express. In other words, implicit learning refers to the incidental or casual, and sometimes seemingly small, acquisition of a given event. It can generate significant future consequences.^42^

Few articles assessed Language,^14^^,^ 35 ^,^ 37 Attention,^23^ Perception^35^ and Visuospatial Skills.^27^^,^ 36 One study^43^ reported PD patients without dementia who exhibited impairment in verbal comprehension, grammatically complex sentence identification, repetitive speech, decreased abstraction capacity, slow processing speed and attention deficit.^44^ There is greater impairment in naming ability and verbal fluency.^8^ Language difficulties may be related to EF, which plays an important role in language. We also found difficulties in understanding grammatically complex sentences, disorders involving communication and repetitive speech.^44^ Attention is impaired in PD, causing reduction of latency in simple and choice reaction times. After dopamine replacement, there is an improvement in the identification of stimuli.^40^ Regarding Visuospatial and Perception skills, these require the recruitment of certain subcortical structures, in addition to the occipital, parietal and frontal lobes.^45^ Deficits in this function in PD correlate with postural instability and gait difficulty.^44^ Sensory-motor coordination also had only 2 instruments for its evaluation.^14^^,^ 20

Only 3 articles^20^^,^ 31 ^,^ 38 used instruments to assess psychiatric symptoms in PD (PANDA, BPRS and Visual Analogue Mood Scale). Some authors^46^ investigated the existence of information on various psychiatric conditions in patients with PD and found that more than 50% of non-motor symptoms are not identified in clinical practice. They observed the prevalence of depression (2% - 31%), psychosis (15% - 75%), anxiety (19% - 67%), sleep disorder (15% - 87%) and cognitive deficits (MCI 18% - 55% and dementia 31%), among others. Psychiatric symptoms were associated with the stage of PD and cognitive impairment of the patient, but not with age, duration of illness, levodopa dose or ‘ON’ or ‘OFF’ stage. Although common, psychiatric changes in PD are not criteria for clinical diagnosis.^47^ These changes can become more disabling than motor deficits and may be a consequence of complications of the pharmacological treatment for the motor symptoms of the disease or as an integral part of the PD clinical manifestations.^48^

Only 2 scales were used for assessing PD Quality of Life (QoL): PDQ-39^49^ and PDQL.^50^ Regarding mood, several scales were used, with use of depression inventory being the most often cited.^14^^,^ 20 ^,^ 21 ^,^ 23 ^,^ 25 ^,^ 27 ^,^ 32 ^,^ 36 ^,^ 37 Together with the QoL scales, these instruments enable a more in-depth examination of the individual and impacts of the disease on their life.

Some of the limitations of the study were the instruments used at different stages of the studies, albeit for the inclusion and/or exclusion criteria and during the pre and post-operative evaluation of patients. Besides the large diversity of instruments, other aspects such as version, validation and cut-off points were also heterogeneous.

The instruments used before and after DBS implantation to assess the cognitive aspects of patients are shown in Table 3. Generically speaking, we could consider them as a possible battery for evaluating the effects of surgery. The literature has shown that most authors consider these instruments sufficient to identify the patient’s diagnostic profile. These aspects are extremely relevant to analyze the results of a study. Differences in each of them may engender results that differ from those expected. These changes range from severely compromised to slightly compromised. Another example is the use of tailored tasks, Verbal Fluency Tasks^13^^,^ 14 ^,^ 16 ^-^ 18 ^,^ 20 ^,^ 21 ^,^ 23 ^,^ 26 ^,^ 27 ^,^ 29 ^,^ 30 ^,^ 32 ^,^ 34 ^-^ 36 ^,^ 38 the n-back task and dual task^51^ rather than standardized tests, such as the Wechsler Adult Intelligence Scale^27^^,^ 35 and Hooper Visual Organization Test.^36^

The results of this review point to the absence of a specific assessment protocol for PD with STN-DBS, revealing extensive variability of instruments used in different studies. However, analysis of each methodology yielded a possible battery for investigating the effects of surgery based on the frequency of use of instruments in the studies. The feasibility of using this battery and its findings should be the focus of future studies to establish a standard for assessment.
